# Automated analysis of ultrastructure through large-scale hyperspectral electron microscopy

**DOI:** 10.1038/s44303-024-00059-7

**Published:** 2024-12-11

**Authors:** B. H. Peter Duinkerken, Ahmad M. J. Alsahaf, Jacob P. Hoogenboom, Ben N. G. Giepmans

**Affiliations:** 1 Department of Biomedical Sciences, University Groningen, University Medical Centre Groningen, Groningen, AV The Netherlands; 2 Department of Imaging Physics, Delft University of Technology, Delft, CJ The Netherlands

**Keywords:** Biological techniques, Imaging, Microscopy

## Abstract

Microscopy is a key technique to visualize and understand biology. Electron microscopy (EM) facilitates the investigation of cellular ultrastructure at biomolecular resolution. Cellular EM was recently revolutionized by automation and digitalisation allowing routine capture of large areas and volumes at nanoscale resolution. Analysis, however, is hampered by the greyscale nature of electron images and their large data volume, often requiring laborious manual annotation. Here we demonstrate unsupervised and automated extraction of biomolecular assemblies in conventionally processed tissues using large-scale hyperspectral energy-dispersive X-ray (EDX) imaging. First, we discriminated biological features in the context of tissue based on selected elemental maps. Next, we designed a data-driven workflow based on dimensionality reduction and spectral mixture analysis, allowing the visualization and isolation of subcellular features with minimal manual intervention. Broad implementations of the presented methodology will accelerate the understanding of biological ultrastructure.

## Introduction

Identification and localisation of the building blocks of cells is crucial to understanding biology. In light microscopy, a toolbox of fluorescent techniques to identify and analyse molecules has revolutionised bioimaging (reviewed in Giepmans et al.)^1^, including in living systems, but typically limited to sub-µm resolution. Electron microscopy (EM) allows the localisation of biomolecules at the resolution of these macromolecules and reveals additional ultrastructural information. Moreover, large-scale and automated EM provide a comprehensive view of tissues at high and low magnifications^2–4^. Mechanisms to automatically identify biomolecules and structures in EM are limited: Manual annotation of large EM acquisitions is intractable, pressuring the use of semi-automatic segmentation tools or citizen science^5,6^. Extending EM analysis with techniques conceptually equivalent to the fluorescence toolbox will allow full analysis of the now automatically acquired large volumes of data and would therefore radically improve its utility in biology.

X-rays are generated during EM imaging and, when collected in high enough quantities and with high energy resolution, allow defining the elemental composition of the ultrastructure (Energy-dispersive X-ray (EDX) imaging)^7^. Elemental imaging is hardly applied in biomedicine because of: (i) the overwhelming presence of carbon and abundance of weakly discriminative low-atomic number elements and (ii) the low EDX collection efficiency leading to long acquisition times for sufficient signal. In addition, if EDX imaging is applied, current practises for analysing the hyperspectral image (HSI) are based on defining the present elements. This may lead to bias by the a priori expected chemical content and may leave part of the hyperspectral image (HSI) uninspected, especially concerning the correlated, stochiometric presence of multiple elements in macromolecules or biostructures. Importantly, EDX acquisitions are rich in data and are co-registered with the electron images, allowing implementations that extend beyond mapping of individual elements.

Data-driven techniques that use the whole HSI, like spectral mixture analysis (SMA) and deep learning-based classification, are popular in remote sensing and materials science and have become the standard for detecting and classifying the materials in an image. In biology, HSI analysis is particularly useful in optical methods, enabling the separation of dyes in fluorescence microscopy and FACS, but its use in biomedical EM remains to be explored^8–11^.

SMA is useful for HSIs that lack annotations, as it enables unsupervised identification of their constituent materials by extracting characteristic spectra, known as endmembers^12,13^. Linear mixture models such as constrained least squares are commonly used to compute the corresponding proportions—also known as abundances—for each pixel^14^. SMA isolates relevant biological features into separate images that could be the EM analogue to fluorescence in optical microscopy.

Here, we extend the use of EDX from acquiring single images to unprecedented large-scale EDX maps at ultrastructural resolution of multiple cells within tissue with optimized acquisition parameters. This yields spectrally rich data that allows data-driven hyperspectral analysis achieving classification of biological structures, which in turn is a first step towards highly automated analysis of large-scale EM. The rich dataset is shared in an open fashion to allow analysts to explore alternative approaches that may yield additional insights. While the current report is a proof-of-concept on an islet of Langerhans in the pancreas and on normal human skin, the resource will be expanded with other tissues in the future.

## Results

### Spatial and elemental context through large-scale EDX

**Fig. 1:**
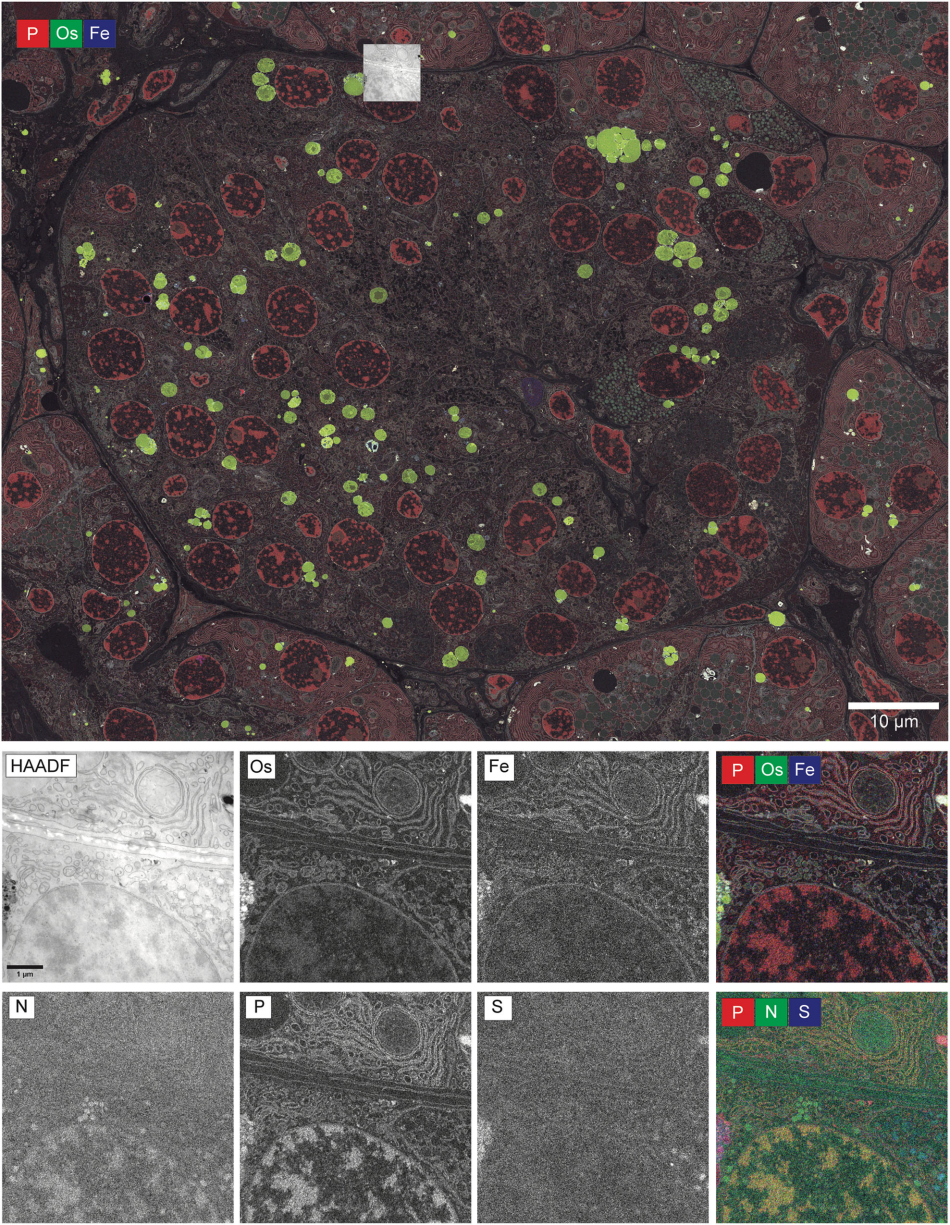
Tissue and elemental context through large-scale EDX imaging. (Top) Phosphorus (P), osmium (Os) and iron (Fe) distribution in an islet of Langerhans. (Bottom) High-resolution zoom-in EM image (HAADF) and elemental maps of osmium (Os), iron (Fe), nitrogen (N), phosphorus (P) and sulphur (S). The HAADF inset (top) illustrates the region from which the zoomed images were retrieved. Each elemental map was processed and scaled individually. All original data files as well as the zoomable EDX images are available at full resolution online.

EDX imaging can differentiate cellular components based on their elemental markup but is typically limited to pre-selected fields of view of an individual cell^7,15^. Therefore, a workflow for large-scale tiled EDX acquisitions was developed and applied to a whole section of an islet of Langerhans. Sufficient spectral richness was ensured by prolonged pixel dwell time and frame accumulation, while long acquisition times were overcome using relatively high beam currents (4–5 nA) and two EDX detectors symmetrically positioned close to the sample. Biological features such as granules, nuclei, lysosomes and ribosome-coated rough endoplasmic reticulum can be clearly distinguished by representing three elements in false colour in the tiled acquisition (Fig. 1). At the same time, the cellular ultrastructure and its elemental composition reveal the distinct elemental mark-up when examined at high resolution (Fig. 1). Lipids are predominantly present in the osmium and iron maps; nitrogen and sulphur highlight protein-packed areas such as granules; heterochromatin and the ribosomal-coated endoplasmic reticulum (ER) are visibly enriched in phosphor. While islets of Langerhans allow different cell types to be distinguished based on the elemental content of their granules, EDX imaging can equally provide additional insights into ultrastructural features of other tissues such as the skin (Figure S1a). In the examples above, we chose which elements to represent, but the large-scale acquisition yields a sufficient amount of data to perform an improved interpretation with more unbiased data-driven spectral mixture analysis.

### Spectral mixture analysis reveals biological features

**Fig. 2:**
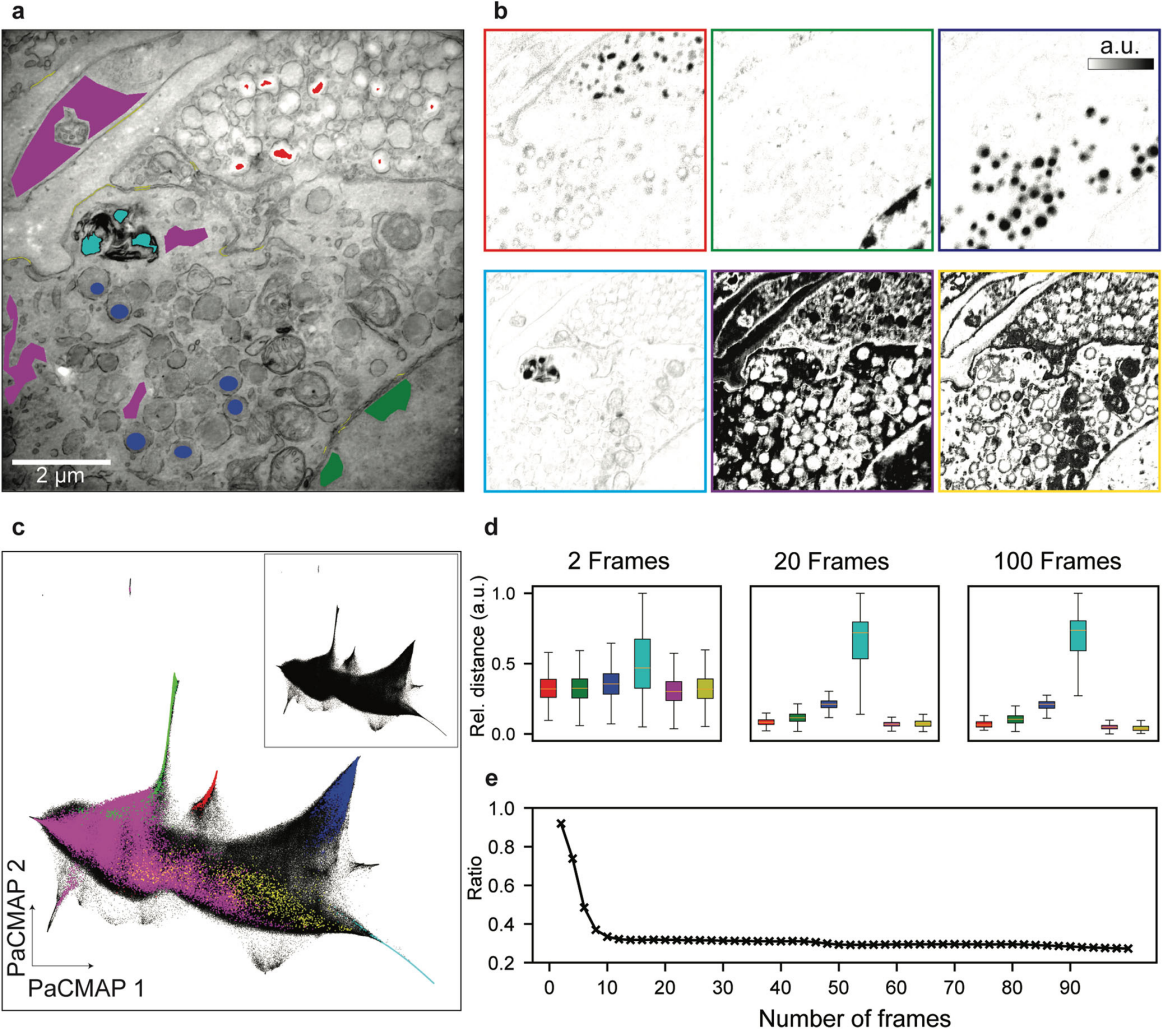
Spectral unmixing and dimensionality reduction of hyperspectral EDX. **a** EM image corresponding to a single EDX acquisition of a 100 nm thick pancreas section. Representative pixels of insulin (red), heterochromatin (green), glucagon (green), a membranous structure (cyan), intracellular space (magenta) and membranes (yellow) are annotated. **b** Abundance maps of the HSI using average spectra from the annotated regions in (**a**) as endmembers. Here, low and high abundances are respectively mapped from white to black and the colour outline corresponds to the annotated feature in (**a**). **c** PaCMAP embedding of the HSI (inset) and labelled according to the annotated pixels in (**a**). **d** Relative Euclidean distance within classes as a function of frame accumulation. **e** Ratio of between the relative Euclidean distance within and amongst classes as a function of frame accumulation. Scale is 2 µm.

Identifying the spectral signatures of biomolecules, exogenous stains, and associated atoms enables visualisation of their spatial distribution. X-ray generation and detection were enhanced by using thicker sections and a tenfold increase to the number of accumulated frames (100) to yield an enriched HSI. Biological features that are expected to be spectrally differential were sparsely annotated by hand, allowing representative spectra to be retrieved (Fig. 2a). Linear unmixing of the HSI, based on the averaged representative spectra, maps the relative computed abundances of each extracted spectrum to the individual pixels (Fig. 2b). While only a subset of pixels was annotated, unmixing yields abundance maps that correspond to the annotated biological features.

Dimensionality reduction techniques, such as manifold learning, highlight patterns and structures in high-dimensional data. In an HSI, dimensionality reduction groups spectrally similar pixels, which is potentially a better alternative for manual annotations in the spatial domain^16^. Local and global structure of the HSI needs to be preserved, which is a hallmark of Pairwise Controlled Manifold Approximation Projection (PaCMAP)^17^. Reducing the HSI to two dimensions with PaCMAP forms a primary asymmetrical data cluster with noticeable separation between the intracellular space (magenta) and membranes (yellow; Fig. 2c). The pixels representing heterochromatin, insulin, glucagon and the multi-layered membranous structure are concentrated in distinct and separate protrusions from the central cluster.

To further reduce acquisition time the minimum number of frames required to maximise the spectral similarity within and between groups was analysed (Fig. 2d and e). At approximately twenty frames the spectral distance from the average spectrum within groups (intra-distances) stabilises, whereas the average ratio between intra- and the inter-distances plateaus at ten frames. Taken together, spectral unmixing in EDX imaging allows the differentiation of biological features where dimensionality reduction can be used to select representative spectra.

### Large-scale spectral mixture analysis (SMA)

**Fig. 3:**
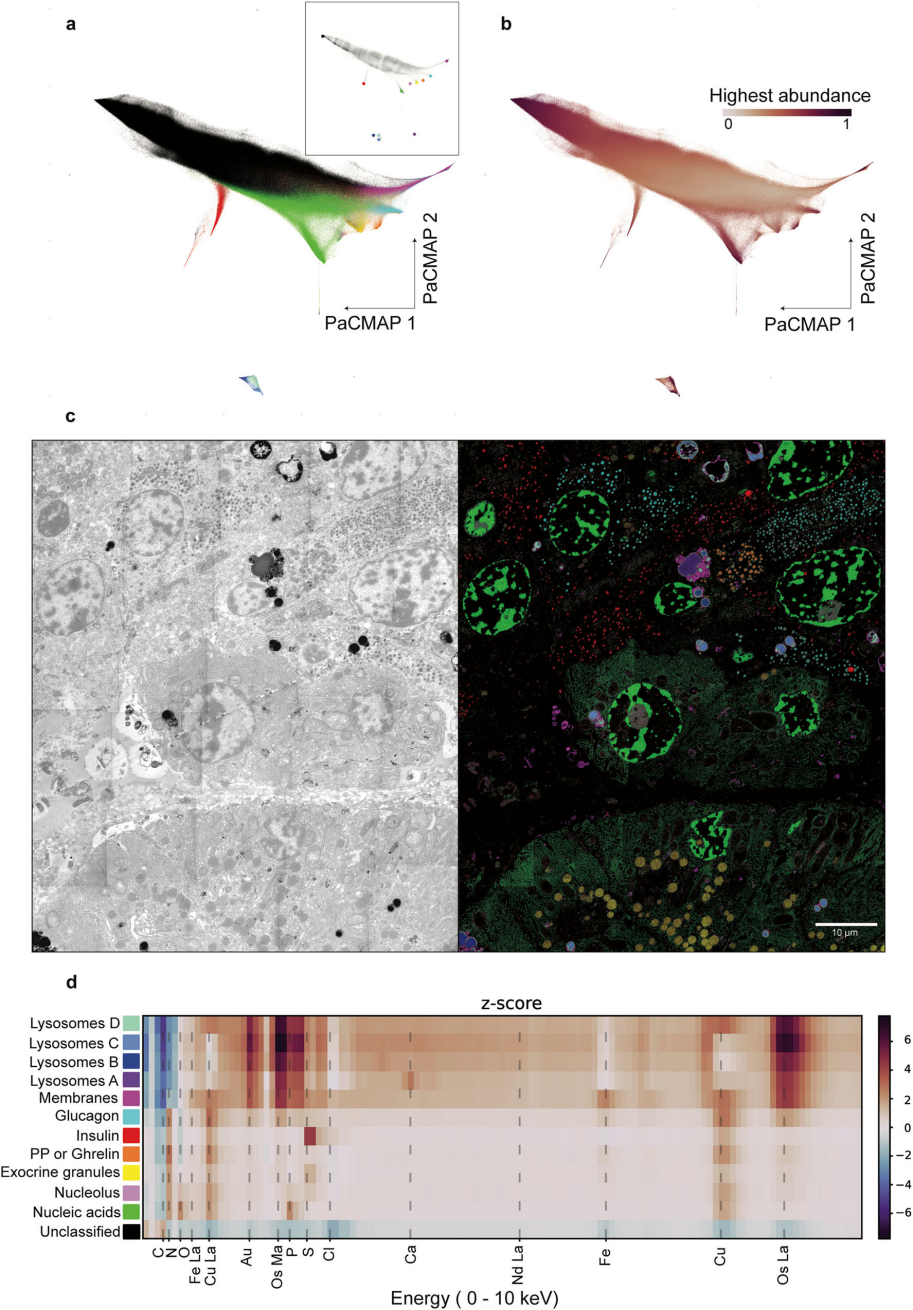
Extraction and identification of endmembers in large-scale EDX. **a** Subsampled (20%) PaCMAP embedding of a large-scale EDX acquisition. Endmembers were identified based on their abundance and separation from the bulk (inset). Labelling of the data points reflects the colour of the most abundant endmember found after linear unmixing. **b** PaCMAP embedding coloured according to the highest abundance value in each pixel. **c** Large-scale EM (High-angle annular dark-field; HAADF) image of a partial islet of Langerhans (left) and spatial representation of the unmixing result with the colour representing the endmember with the highest abundance and the intensity scaled accordingly (right). **d** Heatmap reflecting the per channel Z-score for each extracted endmember. Labels are based on the biological features annotated in (**d**).

Dimensionality reduction through PaCMAP facilitates the identification of the purest (i.e. least mixed) spectra present in the pancreas (Fig. 3a, inset) and the skin (Fig. S1b). However, large-scale EDX comes at the cost of a large amount of data requiring subsampling for dimensionality reduction to be implemented. In addition, the overlapping regions resulting from the tiled image were excluded such that the impact of additional spectral variability introduced by multiple exposures was limited (Fig. S2). The endmembers represent average spectra of groups of similar pixels, which can be used to decompose the HSIs. Spectral unmixing demonstrates the tendency for similar points to group together (Fig. 3a). Furthermore, high abundances towards the extremities of the protrusions from the primary data cluster indicate that the linear mixture model conforms with the structure revealed by the PaCMAP embeddings (Fig. 3b). Spatial mapping and subsequent stitching of the dominant abundances represents a large part of the HSI, with spectrally discernible features corresponding to the ultrastructure of the pancreas (Fig. 3c and Fig. S3) and the skin (Fig. S1c). Similarly, the elemental composition of each endmember can be inspected and compared against the expected biochemical composition of the structures that the abundance maps highlight (Fig. 3d).

### Automated detection and segmentation of EM images

**Fig. 4:**
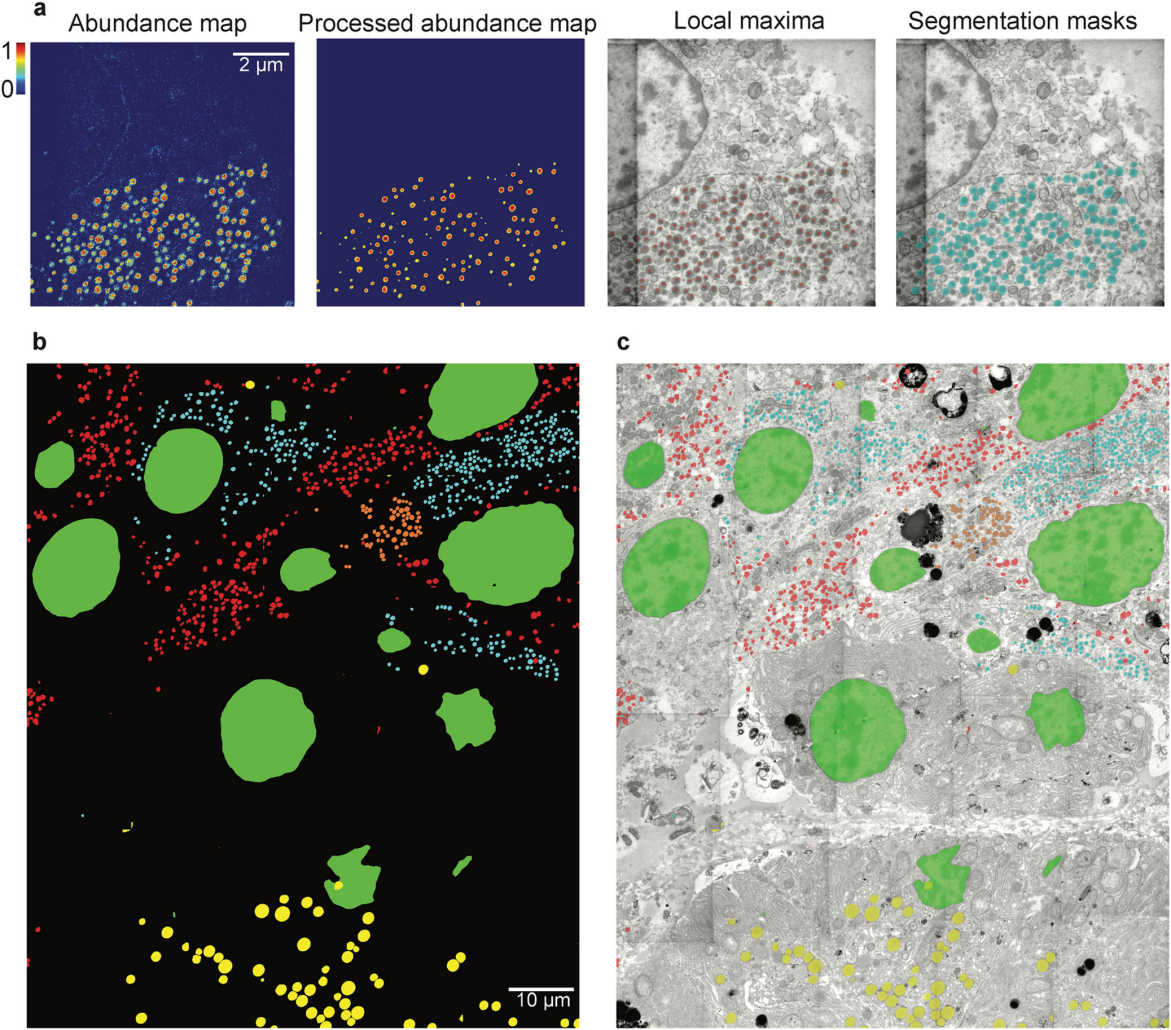
Identification and segmentation of large-scale EM using spectral unmixing and zero-shot learning. **a** Workflow of identification and segmenting structures, from left to right: the abundance map of the selected structure (here glucagon for illustration), the abundance map Gaussian blurred and thresholded, the local maxima from the previous image overlaid on the HAADF image used as point prompts for the SAM model, and the segmentation mask output of SAM. **b**, **c** the workflow in (**a**) applied in large-scale with five structures (insulin, glucagon, nucleic acids, PP or ghrelin, and exocrine granules) resulting in five binary masks shown in a composite image (**b**) and overlaid the large-scale HAADF (**c**).

Convolutional neural network-based methods have been explored in the literature using the electron image alone, with varying degrees of success (reviewed in Aswath et al.)^6^. EDX-based endmembers can aid data-driven automatic segmentation, as shown in a basic implementation using the Segment Anything Model (SAM)^18^. Foundation models for segmentation like SAM achieve impressive performance on many types of medical images, without case-specific model training^19^. Without appropriate prompts, the model segments all objects indiscriminately and without classification. We leverage the structure-specific endmember abundance maps extracted from the HSIs to create spatial prompts for the SAM model (Fig. 4a). Combining these over the large-scale image, results in the clear, automatic detection and segmentation of, in this case, five distinct organelles and biostructures (Fig. 4b and c), providing a compelling illustration of the potential of EDX HSI for unsupervised EM analysis. The average estimated IoU (Intersection over Union) for all structures across the whole dataset is 0.91. The complete results are given in Tables S1 and S2.

## Discussion

EM is increasingly applied on larger scales^2–4^ and volumes^20,21^, complicating the manual analysis of the large amount of the EM images. Elemental imaging has become more popular and dedicated sensitive detectors now allow large-scale mapping, where combinations of elements help to identify ultrastructure as exemplified by EDX imaging. While selection of only a few elements is informative, the hyperspectral nature of EDX imaging at biomolecular resolution, as well as the large-scale, i.e. imaging multiple different structures, lends itself for data-driven analysis.

Manifold learning gives insight into patterns and structures present in the spectral domain. UMAP (Uniform Manifold Approximation and Projection) has similarly been applied to the unmixing of spectral images of artwork^16,22^. However, PaCMAP was favoured for its ability to balance the preservation of global and local structure without the need for hyperparameter tuning^17^. In the resulting embedding, branching appears to reflect a continuum of mixed pixels, with the purest spectra represented at the extremities of the protrusions which can be interactively selected. Thus, PaCMAP may guide segmenting biological features based on spectral characteristics, circumventing the need for manual annotations in the spatial domain. The implementation of PaCMAP for unmixing hyperspectral data of a cellular image expands on the applicability of manifold learning methods in high-dimensional biological data^23–25^.

The extracted endmembers and their abundance maps show a high conformity with ultrastructural categories. The most electron-dense structures are the membranes and lysosomal structures. Indeed, the endmembers highlighting those structures show relatively high osmium, with subtle differences between other elements. The heterochromatin and ribosome-coated rough endoplasmic reticulum show relatively high quantities of nitrogen, phosphorus and oxygen which is in agreement with the concentrated localisation and chemical composition of nucleic acids. The elemental fingerprints of exocrine and endocrine granules are largely determined by the relative abundance of nitrogen, phosphorus and sulphur as shown previously^7^. Note however that increased scattering in electron-dense regions can yield X-rays not originating from the sample, which could explain the proportionally increased copper and aluminium signals. Additionally, more Bremsstrahlung is to be expected across the whole spectrum for regions rich in elements with higher atomic mass^26^. In other tissues other structures are identified, as exemplified in skin, showing the general applicability.

Spectral unmixing is reliant on spectral diversity within the dataset. As such, features that have little spectral variation would be indistinguishable for the current workflow. Therefore, while structures such as the Golgi apparatus, nuclear envelope, mitochondria and the plasma membrane have different biological functions and ultrastructure, they end up in the same endmember class. Incorporation of additional elements, with affinity to specific features - such as lead, neodymium and osmium—has the potential to further expand the spectral variability. Similarly, stains or labels to be developed for EDX imaging containing other elements, for instance, the cadmium-containing Quantum dots^27^, or feature-specific stains similar to MitoTracker in fluorescence are of high interest for further spectral discrimination of organelles that do not uniquely end up in a single endmember class using current sample preparation protocols.

Conventionally processed pancreas was analysed to enable future translation to an existing large EM database^4^. Applying the workflow onto other tissues leads to tissue-specific endmembers, as revealed by the proof-of-principle skin dataset. A wide variety of approaches for image and spectral analysis can be further explored building on our proof-of-concept and data availability: all data is available online for reuse.

An alternative for measuring the elemental composition is elemental energy loss spectroscopy (EELS), which in principle complies with the presented workflow. One advantage of EELS over EDX is its higher spectral resolution. However, we favoured EDX since the range of measured energies is typically broader and acquisitions are less reliant on a priori knowledge of the sample. For a more detailed comparison of analytical modalities applied and pioneered in life sciences see Pirozzi et al.^15^.

The pipeline presented here is applied on large-scale 2D EM, but it would scale linearly to volume EM (vEM)^20,21^ by repeating the current procedure on serial sections. Block-face scanning EMs, in which a new specimen surface is exposed by removing the old after imaging, can in principle be supplemented with EDX analysis as well. However, the unusual heavy staining protocols and underlying structures in the block might skew the acquired spectra. Furthermore, the typically lower acceleration voltages narrow the spectral range that can be recorded. Therefore, the suitability of EDX analysis and subsequent spectral unmixing in block-face scanning EM-based vEM approaches remains to be further explored.

Scaling up EDX is challenging since the acquisitions are three orders of magnitude slower than conventional EM (e.g. Fig. 3 and S1 took ~3 days each). Therefore, it may be more feasible to conduct EDX imaging on representative sets of images as shown here. The resulting abundance maps together with other analysis methods such as SAM have the potential to subsequently be used as ground truth for future segmentation efforts in bare (v)EM images, comparable to fluorescence-guided learning in correlative light and electron microscopy^28^.

How subtle differences in images due to preparation and acquisition in different labs might challenge this procedure remains to be investigated and will likely depend on the model used and the quality of the datasets.

The dedicated instrument used here is not commonly available in life science core facilities, but wide access to instruments is provided through research consortia^29^, enabling broader implementation of the workflow presented here. Future developments will likely target higher X-ray detection yields diminishing dwell time and thus faster acquisition.

In conclusion, routine elemental mapping and unbiasedly analysing the data has the potential to uncover structures that have the same electron density but differ in spectral signal. Given the diverse potential of EDX and the presented pipeline, data-driven spectral analysis of EM will become a principal step in the ongoing high-content and high-throughput revolution that accelerates ultrastructural understanding of the building blocks of life.

## Methods

### Sample preparation electron microscopy

Tissue derived from nPOD (Florida, USA)^30^ and embedded in EPON was reused from a previous study where details on sample preparation and ethical approval are included^4^. Briefly, two different samples (nPOD #6126, 6226) were aldehyde fixed. The tissue was sectioned at ~50 µm (HM 650 V, Microm), post-fixed in 1.5% osmium tetroxide (19114, EMS) reduced with 1.5% potassium ferrocyanide (4984, Merck), followed by dehydration using a graded series of ethanol and subsequently MgSO_4_ dried acetone, EPON infiltration and polymerisation overnight. Embedded tissue was sectioned (UC7, Leica) at a thickness of 50 nm (Fig. 1) or 100 nm and placed on formvar-coated single-hole copper grids (G2010-Cu, EMS). The skin sample was additionally post-stained using 4% neodymium acetate in milliQ for 30 min at room temperature^31^. Prior to imaging, a 5 nm thick layer of carbon was sputter-coated (ACE600, Leica) on the formvar side of grids to limit charging of the samples and improve stability of image acquisition. The skin sample was re-used from a previous study (dataset 2) and only re-sectioned^32^.

### Energy-dispersive X-ray imaging

Grids were mounted in a low background double-tilt scanning transmission electron microscopy (STEM) specimen holder optimised for EDX imaging (4022 190 50334, Thermo Fisher Scientific). Samples were imaged at 80 kiloelectronvolt (keV) on a Thermo Scientific Talos F200i STEM equipped with an S-FEG. High-angle annular dark-field (HAADF) imaging was conducted using a HAADF detector and two Bruker X-Flash 6T-100 EDX detectors for simultaneous EM and EDX imaging. The HAADF images, referred to as EM images in the main text, were taken with a camera length of 205 mm and the EDX detectors were set to capture up to 20 keV using 5 eV bins. A 150 µm condenser aperture was used in nanoprobe mode with a typical screen current of 4–5 nanoamperes (nA) to maximise X-ray generation. Each tile was the product of 10 (Fig. 1), 20 (Figs. 3 and S1), or 100 (Fig. 2) frames of 2048 by 2048 pixels with 4096 energy channels each, acquired with a pixel dwell time and size of 100 µs and 4 nm, respectively. Thus, a single tile in which 20 frames are accumulated takes approximately 2.5 h, for a total of ~3 days to acquire a 40 × 48 µm area of 30 tiles (Fig. 3). Drift correction between frames was provided by the acquisition software and based on cross-correlation between the simultaneously acquired HAADF images. Prior to STEM-EDX acquisition, the selected areas were pre-irradiated in TEM mode at 80 keV until no notable brightening was observed. Acquisition was done using Thermo Scientific Velox which also provided the used elemental maps. Thermo Fisher Scientific MAPS software was used for mosaic acquisitions.

### HSI pre-processing

The pre-processing procedure is based on ref.^33^ and is aimed at denoising the HSI and reducing its sparsity. Each individual HSI of dimensions 2048 × 2048 × 4096 is binned spatially and spectrally to a reduced dimension of 1024 × 1024 × 250. The first 96 channels are calibration artefacts and were discarded prior to binning. Then, a spatial mean kernel is applied to each of the 250 images corresponding to the binned spectral bands.

### Extraction of endmembers

The pre-processed HSI is flattened to a 1024^2^ × 250 array. Similarly, Gaussian blurred copies of the HSI are generated with radii 1, 3, and 5, and σ = 2, to account for spatial correlations. The features from the base flattened HSI and the Gaussian filtered versions are concatenated to a 1024^2^ × 1000 array, which is reduced to two dimensions using PaCMAP^17^. Dimensionality reduction of large-scale EDX was based on a subsample (20%) of all pixels present in the mosaic, with the overlapping regions excluded. A 2D histogram is computed from the 2D embeddings and local maxima that are at the extremities of protrusions from the primary cluster of the histogram are selected using an interactive tool. The average spectrum of pixels in a bounding box over each local maximum is designated as an endmember^16^.

### Spectral unmixing and visualisation

The abundances of the endmembers are estimated using non-negative least squares. The colour visualisation of abundance maps (Fig. 3c) is made by colour-coding each pixel according to the most abundant endmember and scaling the intensity according to the endmember’s abundance in that pixel, i.e. a pure pixel will have a solid colour. All coefficients from non-negative least squares were scaled by the maximum coefficient in all abundance maps across the mosaic to ensure consistent colour visualisation. The grayscale levels of all HAADF pancreas images were made comparable by lowering the pixel values of each tile such that the medians are similar to the lowest median of all tiles. Stitching the HAADF images was conducted using the TrakEM2 plugin in FIJI^34,35^. The HAADF and elemental images were all individually min/max normalised, where the bottom and top 1% (0.5% for Fig. 2) of the whole image were allowed to saturate. The elemental and abundance maps were subsequently stitched using the HAADF stitching as a template. Prior to min/max normalisation, each individual elemental map was subjected to a Gaussian blur (σ = 1 px).

### Detection and segmentation

The SAM segmentation masks were produced using the pre-trained model ViT-H^18^. The spatial point prompts were generated by finding the local maxima of a thresholded and Gaussian filtered version of the abundance maps corresponding to five structures. A standard deviation of 4 was used for the filters, and a threshold of 0.5 for the nucleic acid abundance maps, and 0.4 for the remaining structures. Furthermore, the masks of the nucleic acids are dilated and eroded with a 70-pixel radius disk element. Note that in the case of nucleic acid abundance maps, the prompting workflow produces prompt points in heterochromatin regions within nuclei, which in aggregate leads to effective segmentation of nuclei.

## Supplementary information

Supplementary Information

## Data Availability

The raw data as well as the processed data are available at full resolution via www.nanotomy.org. The data has been submitted to IDR (https://idr.openmicroscopy.org/). The code used for the analysis of the data is publicly available via github.com/amjams/Hyperspectraledx.

## Code availability

The underlying code for this study is available in GitHub and can be accessed via this link: github.com/amjams/Hyperspectraledx.
