# Supplementary material for: Automated analysis of ultrastructure through large-scale hyperspectral electron microscopy
Source: Npj Imaging. 2024 Dec 11;2:53. doi: 10.1038/s44303-024-00059-7 (PMC12118689; doi:10.1038/s44303-024-00059-7)
Supplement: Supplementary file 1 — Supplementary Information [file 44303_2024_59_MOESM1_ESM.pdf]

Supplementary results

Automated analysis of ultrastructure through large-scale hyperspectral electron microscopy

B.H. Peter Duinkerken[1,3], Ahmad M.J. Alsahaf[1,3], Jacob P. Hoogenboom[2] & Ben N.G. Giepmans[1,4*]

Affiliations

[1]Department of Biomedical Sciences, University Groningen, University Medical Center Groningen, Groningen, 9713 AV, The Netherlands

[2]Department of Imaging Physics, Delft University of Technology, Delft, 2628 CJ, The Netherlands

[3]These authors contributed equally

[4]Lead contact

[*]Correspondence: b.n.g.giepmans@umcg.nl

Contents

1. **Figure S1** Large-scale EDX and unmixing of skin ultrastructure
2. **Figure S2** Tiled acquisition affects EDX data
3. **Figure S3** Colored visualization of abundance maps
4. **Table S1** Estimated IoUs of SAM
5. **Table S2** Summary of the estimated IoUs of SAM

Note that all data is available at www.nanotomy.org

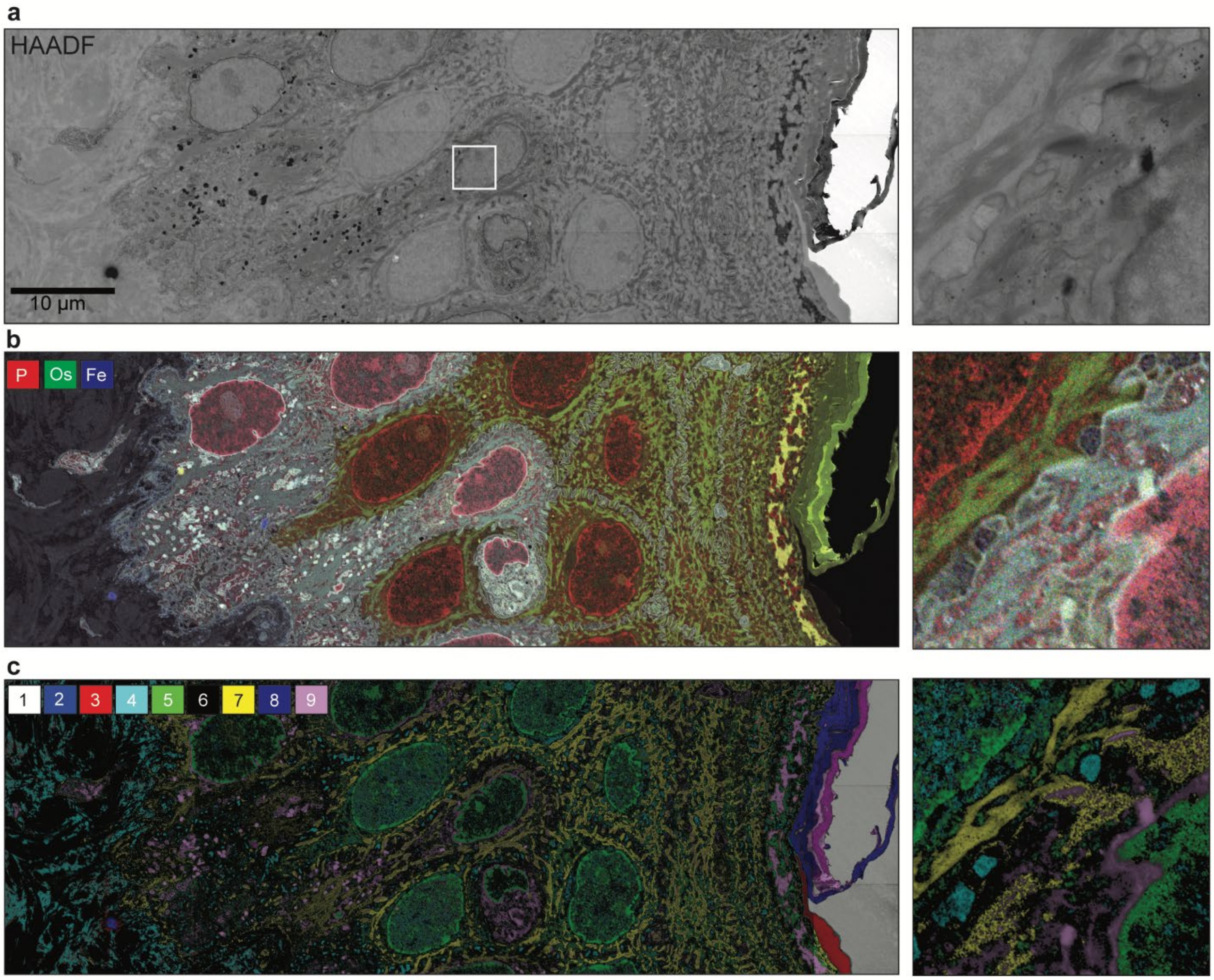

Figure S1 Large-scale EDX and unmixing of skin ultrastructure. (a) EM image (HAADF) of healthy skin data. (b) Distribution of phosphorus (P), osmium (Os) and iron (Fe) distribution in skin. (c) Spectral unmixing primarily highlights EPON (1), separate stratum corneum segments (2, 3, and 9) extracellular matrix (4), heterochromatin (5), intermediate filaments (7), and membranes and pigments (8).

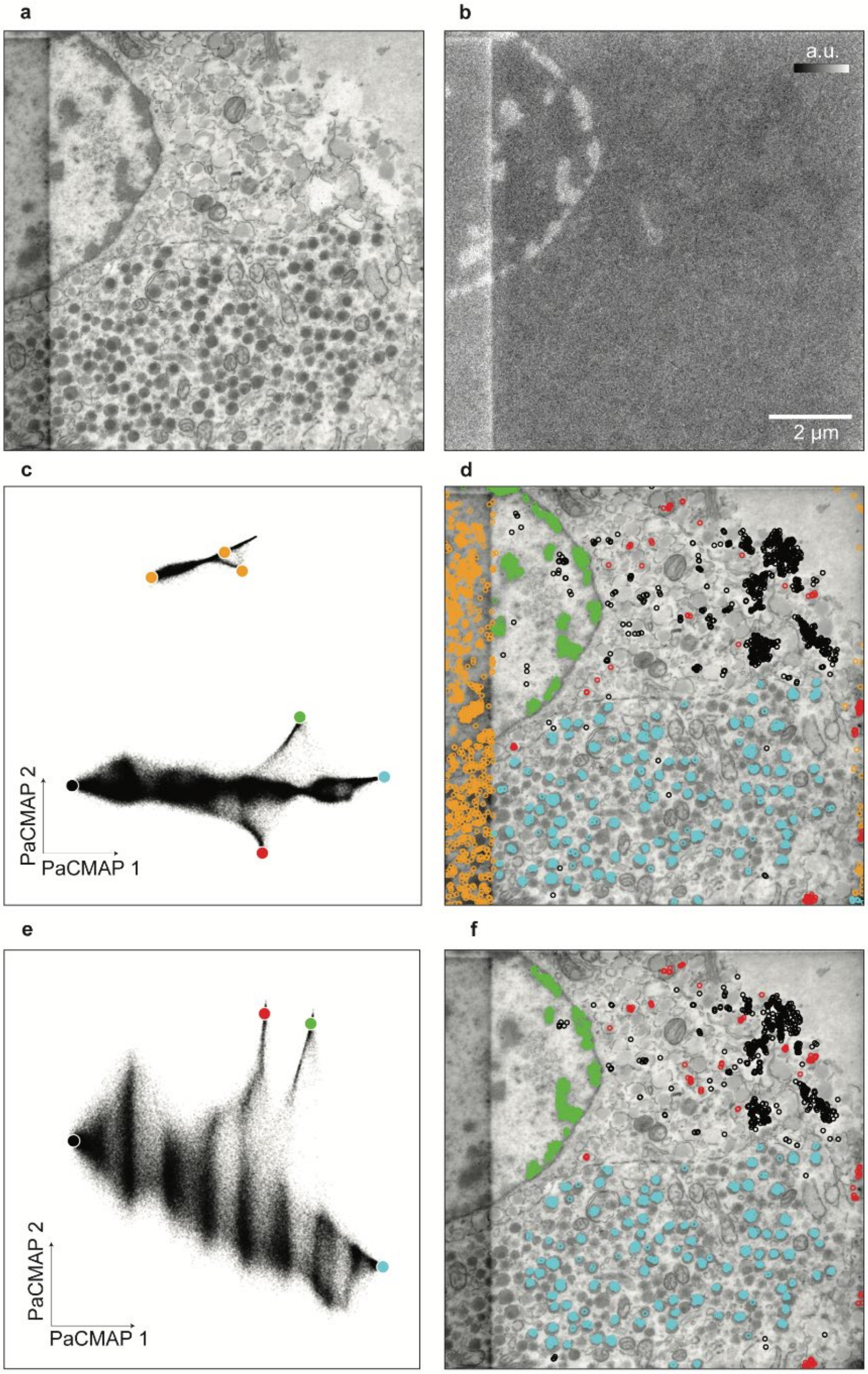

Figure S2 Tiled acquisition affects EDX data. The effect of multiple exposures of the same area is seen in both the EM image (a), and the EDX data, of which the oxygen map is shown (b). The PaCMAP embedding of a 20% random sample is shown for both cases when the multi-exposed pixels are included (c) and excluded (e). The respective localizations of the selected endmember regions are shown in (d) and (f).

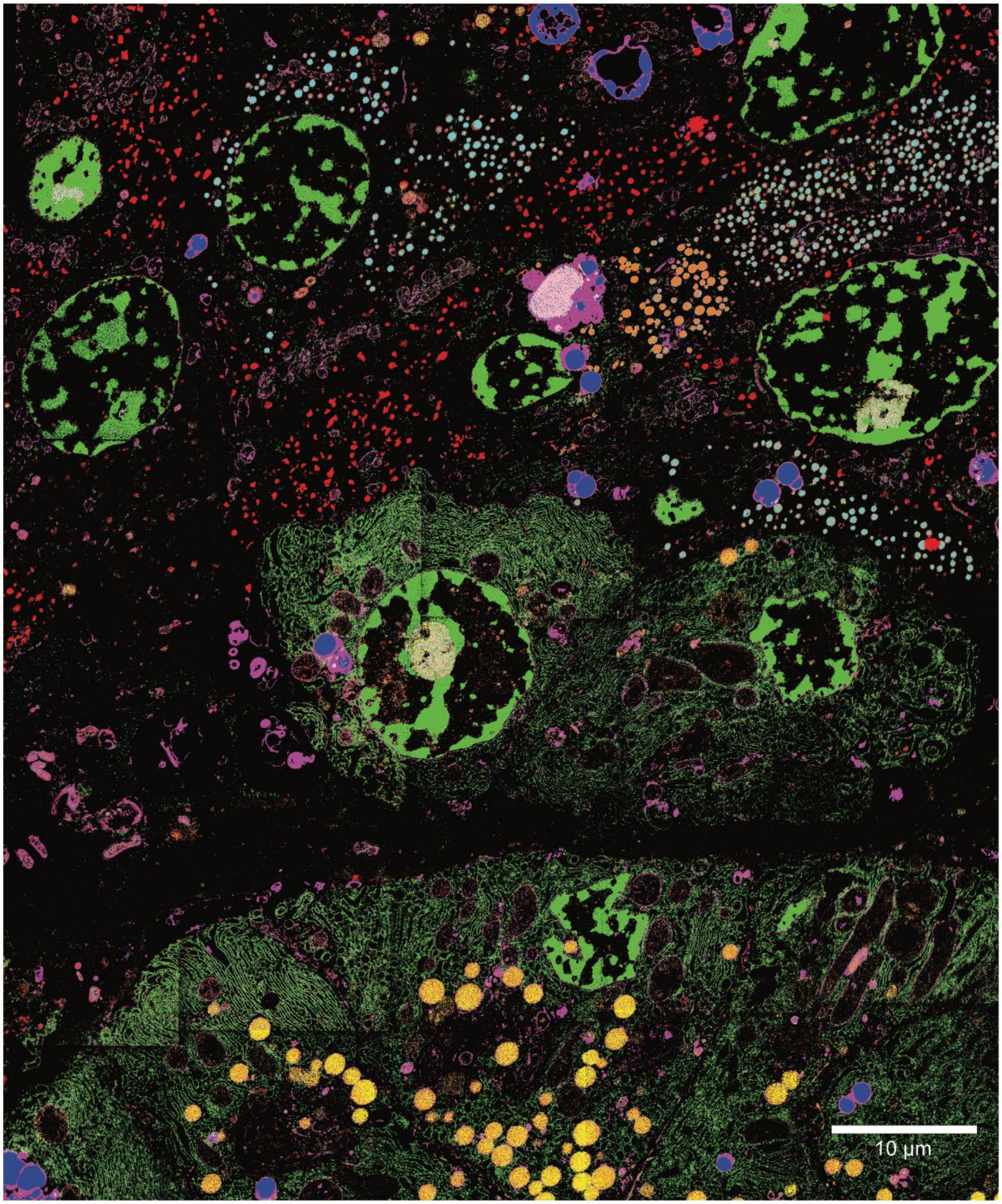

Figure S3 Colored visualization of abundance maps. Spatial representation of the spectral unmixing result with the colour representing the endmember with the highest abundance.

Table S1 Estimated IoUs of SAM. Data reported per structure per tile of the pancreas dataset. Empty cells indicate no instances of the structure were found in the tile.

	Insulin	Nucleic acids	Exocrine granules	PP or Ghrelin	Glucagon
00	0,94	0,93			0,95
01	0,92	0,77	1,0		0,95
02	0,93	0,93	0,92		0,95
03	0,94	0,85			0,95
04	0,93	0,89	0,90		0,96
05	0,94	0,86			0,95
06	0,93	0,76	0,92		0,95
07	0,94	0,85	0,92	0,97	0,95
08	0,95	0,83	0,94	0,98	0,95
09	0,92	0,90	0,93		0,96
10	0,94	0,80	0,98		
11	0,94	0,80			
12	0,91	0,89	0,62		
13	0,91	0,92	0,99	0,96	0,96
14	0,92	0,74			0,96
15	0,93				0,91
16	0,92	0,82			
17		0,73	0,99		
18	0,71	0,83			
19		0,87			0,94
20	0,95				0,81
21	0,94	0,36	0,97		
22		0,84	0,98		
23		0,80	1,0		
24		0,95			
25	0,95				
26			0,99		
27			0,99		
28			0,98		
29	0,73		0,98		

Table S2 Summary of the estimated IoUs of SAM. Averaged data for all tiles of the pancreas dataset. Count refers to the number of tiles that contain segmented instances of the corresponding structure.

	Insulin	Nucleic acids	Exocrine granules	PP or Ghrelin	Glucagon
count	22	23	18	3	15
mean	0,91	0,82	0,94	0,97	0,94
std	0,063	0,12	0,087	0,0080	0,038
min	0,71	0,36	0,62	0,96	0,81
25%	0,92	0,80	0,93	0,97	0,95
50%	0,93	0,84	0,98	0,97	0,95
75%	0,94	0,89	0,99	0,97	0,95
max	0,95	0,95	1,0	0,98	0,96
